# Emotional Voice Processing: Investigating the Role of Genetic Variation in the Serotonin Transporter across Development

**DOI:** 10.1371/journal.pone.0068377

**Published:** 2013-07-08

**Authors:** Tobias Grossmann, Amrisha Vaish, Janett Franz, Roland Schroeder, Mark Stoneking, Angela D. Friederici

**Affiliations:** 1 Early Social Development Group, Max Planck Institute for Human Cognitive and Brain Sciences, Leipzig, Germany; 2 Department of Developmental and Comparitive Psychology, Max Planck Institute for Evolutionary Anthropology, Leipzig, Germany; 3 Max Delbrück Center for Molecular Medicine, Berlin, Germany; 4 Department of Evolutionary Genetics, Max Planck Institute for Evolutionary Anthropology, Leipzig, Germany; 5 Department of Neuropsychology, Max Planck Institute for Human Cognitive and Brain Sciences, Leipzig, Germany; Vanderbilt University, United States of America

## Abstract

The ability to effectively respond to emotional information carried in the human voice plays a pivotal role for social interactions. We examined how genetic factors, especially the serotonin transporter genetic variation *(5-HTTLPR)*, affect the neurodynamics of emotional voice processing in infants and adults by measuring event-related brain potentials (ERPs). The results revealed that infants distinguish between emotions during an early perceptual processing stage, whereas adults recognize and evaluate the meaning of emotions during later semantic processing stages. While infants do discriminate between emotions, only in adults was genetic variation associated with neurophysiological differences in how positive and negative emotions are processed in the brain. This suggests that genetic association with neurocognitive functions emerges during development, emphasizing the role that variation in serotonin plays in the maturation of brain systems involved in emotion recognition.

## Introduction

The human voice is the most important sound in our environment. Our ability to analyze and effectively respond to information carried in the voice plays a pivotal role for social functioning. The voice not only carries speech information but it can also be seen as an “auditory face” that enables us to recognize individuals and their emotional states [1]. Emotion produces changes in respiration, phonation, and articulation, which in turn determine the acoustic signal [2]. Emotional tone of voice or emotional prosody comprises different acoustic parameters such as time structure, loudness, roughness, and fundamental frequency. The emotion that is expressed by a speaker is characterized, across cultures, by universal properties of these parameters [3], [4]. Adult listeners can reliably and rapidly recognize different emotions on the basis of these vocal cues [5]. Furthermore there is evidence that emotional prosody is processed non-voluntarily [6], and that the specific acoustic patterns observed in humans in response to certain emotions are very similar to those observed in primates [7]. Recognizing emotional expressions during social interactions permits us to detect another person’s emotional state or reactions, and can provide cues on how to respond appropriately in different situations [8]. While there has been extensive work investigating the brain basis of voice processing in general [9], [10] and emotional voice processing in particular [11], [12], only very little is known about how individuals differ in processing emotional information carried in the voice and what factors may contribute to such differences.

One successful approach to studying variability in emotional sensitivity has been to examine in adults how genetic variation within specific neurotransmitter systems impacts differences in the brain responses to emotional stimuli [13]. The neurotransmitter serotonin (5-hydroxytryptamine; 5-HT) has been shown to play a major role in emotional and social behavior [14]. Specifically, there are a number of studies that revealed effects of genetic variation of the Serotonintransporter (*SLC6A4*/*5-HTTLPR*) on the processing of emotional information conveyed by visual stimuli [15]–[17]. This functional polymorphism (*5-HTTLPR*) in the regulatory regions of the serotonin transporter gene has a short (s) and a long (l) allele (14- and 16-repeat alleles, respectively) that alters promoter activity: the s variant produces significantly less serotonin transporter mRNA and protein than the l variant, resulting in higher concentrations of serotonin in the synaptic cleft. Individuals carrying the s allele appear to have increased anxious temperament, resulting in an elevated risk to develop depression [18], [19]. On the neural level, healthy non-depressed adults carrying the s allele showed an increased amygdala response to threatening stimuli such as fearful and angry facial expressions [15]. Furthermore, structural analyses revealed reduced gray matter in s allele carriers in anterior cingulate and amygdala, and during the processing of facial expressions signalling threat, these regions showed less functional coupling in carriers of the s allele [17]. All this prior work has been focused exclusively on visually presented emotions, while similar work on the processing of emotions from the voice is lacking.

Another critical aspect to consider when assessing the association between genetic variation in the serotonergic system and emotional brain processes is the role that genetic variation may play in development [20]. More specifically, in animal studies it has been shown that transient inhibition of 5-HTT during early development produce abnormal emotional behavior in adult mice, pointing to the importance that serotonin has in the maturation of brain systems that are involved in emotional functioning later in life [21]. In humans, it has been argued that studying genetic association effects in infancy provides the opportunity to examine gene effects at a time in development when genetic association might be more robustly demonstrated because effects of postnatal experience are still relatively small [22]. This argument holds in particular for those social and cognitive processes that emerge early and require little or no experience to develop [23]. For the processing emotional facial expressions, there is evidence from recent work with 7-month-old infants, showing that brain responses to emotional faces vary as a function of *5-HTTLPR* [24]. Specifically, in this study, infants homozygous for the short allele showed less sensitive brain responses to happy facial expressions, suggesting that genetic variation at this locus is associated with differences in emotional face processing early in development. However, it has also been suggested that genetic variation might have no effects or different effects in infancy when compared to adulthood, because the effects of genetic variation observed in adulthood are an outcome of developmental processes [25], [26]. Indeed, it has been shown that the heritability of cognitive abilities increases with age from childhood to adolescence, supporting the notion that some genetic association effects are not independent of development [27]. This suggests that it is important to investigate the impact that genetic variation has across development to better understand the nature and origins of differences in emotion processing.

In the present study, we addressed the question of whether and how genetic variation in the serotonin transporter is associated with differences in processing emotional information carried by the human voice across development. We thus examined the effects of *5-HTTLPR* on the processing of emotional tone of voice (emotional prosody) in adults and infants by using event-related brain potentials (ERPs). We had adults and infants listen to neutral, positive (happy), and negative (angry) voices. We measured ERPs for two reasons, first, because it allows for the precise measurement of the timing of brain processes and second, because ERPs are the method most readily used to study and compare brain processes in infants and adults [28]. Based on prior work [11], [29], [30], we predicted that adults’ brain responses will show differences between emotions during later stages (N400) of processing over anterior brain regions, reflecting semantic-level processes associated with the cognitive evaluation of the emotions [11]. On the basis of prior work with infants, we predicted that 7-month-old infants will also discriminate between emotions, but, unlike adults, they will show early perceptual-level ERP differences distinguishing between emotions. Given that a semantic N400 in language processing cannot yet be observed at the age of 7 months, but only in the second year of life [31], it is likely that infants’ responses to the different emotions will only be observed in perceptual ERP effects. With regard to the association with genetic variation in *5-HTTLPR* we therefore tested between three possibilities: genetic variation is associated with differences (*i*) in early perceptual processing in infants, (*ii*) in later cognitive processing in adults, and (*iii*) in both early perceptual processing in infants and later cognitive processing in adults.

## Experimental Methods

### Participants

The adult sample consisted of 57 participants (28 females, *M = *25 years, *Range = *19 to 32 years). The adult participants were paid for their participation. The adult participants had no prior history of psychiatric illness. The infant sample consisted of 48 7-month-old infants (24 females, *M = *221 days, *Range = *216 to 226 days). An additional five 7-month-olds were tested but not included in the final sample due to fussiness. All infants were born full-term (37–42 weeks gestation) with normal birthweight (>2500 g). All adult participants and parents of the infants gave written informed consent before the study. This study and the written consent procedure were approved by the Ethics committee of Leipzig University Medical School.

### Stimuli

The stimulus material consisted of previously used and validated material consisting of 74 semantically neutral German verbs [29], [32]. A female speaker produced all words with happy, angry, and neutral prosody. Words were taped with a DAT recorder and digitized at a 16-bit/44.1 kHz sampling rate. Analysis of the speech stimuli was performed with the software program “Praat Speech Processing Software” (Boersma & Weenik, Institute of Phonetics Sciences of the University of Amsterdam). The following acoustic parameters were evaluated: (a) mean duration in ms (neutral = 784.04, *SD* = 97.64; happy = 862.22, *SD = *97.69; angry = 932.11, *SD = *118.88), (b) mean fundamental frequency in Hz (neutral = 250.22, *SD* = 21.25; happy = 340.96, *SD = *52.57; angry = 247.43, *SD = *18.89), and (c) mean intensity in dB (neutral = 66.24, *SD* = 4.32; happy = 67.84, *SD = *4.86; angry = 67.33, *SD = *6.51). These three parameters were then used to compare acoustic differences across the three emotions. Means were compared using *t-* tests, which revealed that angry stimuli were significantly longer in their duration than happy stimuli (*t = *5.51, *p*<0.001), and happy stimuli were longer than neutral stimuli (*t = *9.93, *p*<0.001). Furthermore, mean fundamental frequency was significantly higher for happy stimuli than for angry (*t = *17.7, *p*<0.001) and neutral (*t = *15.5, *p*<0.001) stimuli, whereas neutral and angry stimuli did not differ in their fundamental frequency (*t = *0.95, *p*>0.34). The three stimuli did not differ with respect to their mean intensity.

### Procedure

The adult participants sat in a dimly-lit, sound-attenuated, and electrically-shielded room facing a computer screen. Adults were instructed to sit still and watch the screen but no task was given in order to ensure that the data could be compared between adults and infants. Infants were seated on their mother’s lap in a dimly lit, sound-attenuated, and electrically-shielded room. Mothers were listening to music via headphones during the experimental session so that they could not hear the acoustic stimuli presented to their infant. The session continued until the infant had attended to the maximum number of trials (222) or got tired of the experiment. All adult participants listened to the maximum number of trials. The experimental session consisted of consecutive presentations of 74 words from each emotional prosody category (happy, angry, and neutral). Stimuli from the different emotional categories were randomly distributed over the session with no more than two stimuli of the same category occurring consecutively. The inter-stimulus interval varied randomly between 1500 and 2000 ms. During the presentation of the acoustic stimuli, an abstract screensaver without social stimuli was presented to adults and infants on a computer screen placed at a 60 cm distance in order to reduce eye movement artefacts.

### EEG Measurement and Data Analysis

In adults the EEG was recorded from 67 Ag/AgCl electrodes, referenced to the left mastoid. In infants the EEG was recorded with Ag-AgCl electrodes from 19 scalp locations of the 10–20 system, referenced to Cz. Horizontal and Vertical EOGs were recorded bipolarly. Sampling rate was at 250 Hz. EEG data was re-referenced to the algebraic mean of the left and the right mastoid electrodes, and band-pass filtered with 0.3 to 20 Hz (1501 points). Data were baseline corrected by subtracting the average voltage in the 200 ms baseline period from each post-stimulus data point. For elimination of artifacts caused by eye and body movements, EEG data for the whole trial were rejected off-line whenever the standard deviation within a 200-ms gliding window exceeded 80 µV for the vertical or horizontal electro-oculogram and 50 µV at any electrode. The mean number of artifact-free trials in adults was 63.4 (*SD = *8.8) for happy and 63.6 (*SD = *9.3) for angry voices. The mean number of artifact-free trials in infants was 36.6 (*SD = *15.3) for happy and 36.1 (*SD = *15.1) for angry voices. Mean ampltiude ERP effects were analyzed by repeated measures ANOVAs with within-subject factors of emotion (happy, angry), hemisphere (left, right), and the between-subjects factor *5-HTTLPR* genotype (long/long, long/short, short/short). In adults and infants time windows were chosen by visual inspection around the peaks of the major ERP components (N100-P200-N300/400 complex) at frontal and central electrodes commonly investigated in auditory processing [33], [34]. In order to control for general effects of speech/language processing when hearing a speech sound and to isolate the effects of emotional tone of voice, the ERP mean amplitude during these time windows was calculated by subtracting neutral voices from both happy and angry voices. This subtraction method serves as a fom of baseline correction by changing the baseline from brain activity without hearing a sound to brain activity evoked by hearing a speech sound. A similar substraction method was successfully employed in a behavioral study on biased attention and its association with the *5-HTTLPR* variation using visual stimuli in adults [35].

### Samples and DNA Extraction

Buccal samples where collected from each adult and infant with informed consent (from a parent in the case of the infants). Swabs were placed in a lysis buffer and DNA was extracted as described previously [36].

### DNA Amplification and Genotyping

PCR-amplification for genotyping the rs4795541 *5-HTTLPR* indel polymorphism was carried out in an MJ Research Thermal Cycler (MR Research, Waltham, MA, USA). Each 25 µl PCR reaction consisted of an initial DNA denaturation and Taq activation step at 95° for 15 min followed by 34 repeated cycles of denaturation at 95° for 30 sec, an annealing step for 30 sec min at 66° and extension at 72° for 45 sec. The reactions included 20 ng of template DNA, 1× PCR buffer mix 1 (ABgene, Hamburg, Germany), 500 µM dNTPs (Amersham Biosciences, Uppsala, Sweden), 400 nM of each primer (Biotez, Berlin, Germany) and 1.25 unit of Extensor Long PCR Enzyme (ABgene, Hamburg, Germany). Primers (*5-HTTLPR*; Forw.: TCCTCCGCTTTGGCGCCTCTTCC and Rev.: TGGGGGTTGCAGGGGAGATCCTG) were those described previously (Wendland et al., 2006). Following amplification products were electrophoresed through a 2% SeaKem LE gel (Cambrex, Rockland, ME, USA) for 1.5 hours at 120V and stained with ethidium bromide, with a 512 bp product corresponding to the long (l) allele and a 469 bp product corresponding to the short (s) allele. One limitation of the current approach is that we did not include in our genotyping protocol two more recently described variants that have been identified in the long version of *5-HTTLPR* (L_G_ and L_A_), with the L_G_ allele showing similar levels of serotonin transporter expression as the short allele (Hu et al., 2005). However, individuals carrying the L_G_ variant of the long allele are very rare (approximately 10%) and it is thus unlikely that this impacted the current results. The distribution of genotypes at the *5-HTTLPR* polymorphism was as follows: long/long (15), short/long (26), short/short (16) in the adult sample, and long/long (19), short/long (21), short/short (8) in the infant sample. Genotype frequencies did not deviate significantly from Hardy-Weinberg expectations. In our adult sample, in order to examine potential variation in personality traits associated with genotypic variation, we administered the NEO-Five Factor Inventory (NEO-FFI) [37], a 60-item measure of the big five personality traits: extraversion, agreeableness, conscientiousness, neuroticism, and openness to experience.

## Results

### Adults

**Figure 1:**
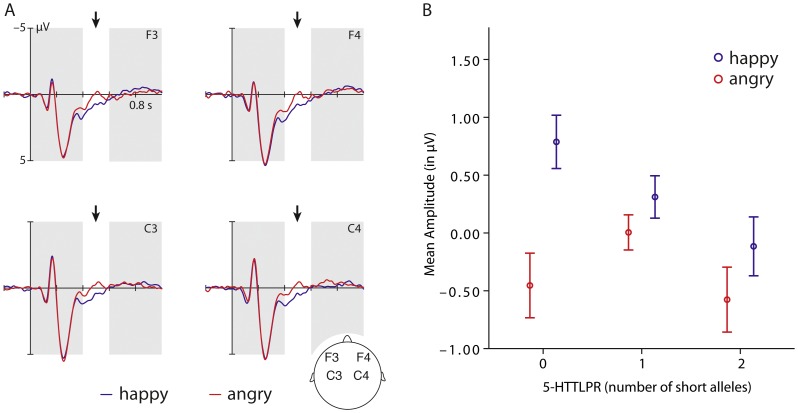
This figure shows the grand-average of the ERP responses to happy (blue) and angry (red) tone of voice at frontal and central electrodes in adults. (A) The graph (B) on the right side shows the mean amplitude of the ERP response to happy (blue) and angry (red) voices between 400 and 600 ms according to the genotype groups.

As shown in Figure 1A, the ERP analysis performed on the grand average data for the adult group revealed different effects as a function of emotion. In a time window between 400 and 600 ms (*F* [1], [54] = 30.57, *p*<0.001, partial *η^2^* = 0.361) angry voices elicited a more negative-going waveform when compared to happy voices. This difference had its maximum at anterior (frontal and central electrode) sites. There were no ERP differences between emotions during earlier time windows. Our analysis further revealed an interaction between the factors emotion and *5-HTTLPR* genotype (*F* [2], [54] = 7.61, *p* = 0.001, partial *η^2^* = 0.22) during that time window, suggesting that genetic variation at this locus was associated with differences in the ERP responses to emotional tone of voice. As shown in Figure 1B, our analysis revealed that carriers of the short allele of *5-HTTLPR* showed a significantly decreased differentiation between happy and angry tone of voice (*F* [2], [54] = 5.66, *p* = 0.006): posthoc tests showed that the difference between the ERP response to happy and angry voices was signigicantly greater in the long/long genotype group than in the short/long genotype group (*p* = 0.017) and in the short/short genotype group (*p* = 0.002). More specifically, for happy tone of voice the number of short alleles was associated with a linear decrease in the amplitude of this ERP component (see Figure 1B), statistically reflected in a negative correlation between the number of short allele and the ERP response to happy voices (*r* = -0.339, *p* = 0.01). Moreover, for angry tone of voice there was a non-linear (inverted u-shaped) relation between the number of short alleles and the amplitude of this ERP component in response to an angry tone of voice (see Figure 1B). Individuals homozygous for the short allele (*M* = -0.58, *SE* = 0.27) and individuals homozygous for the long allele (*M* = -0.45, *SE* = 0.28) showed a more negative-going ERP response than the heterozygous individuals (*M* = 0.005, *SE* = 0.15). However, posthoc comparisons revealed no statistically significant differences between genotype groups with respect to the ERP response to angry voices. Importantly, there were no differences in personality traits such as neuroticism or extraversion as measured by the NEO-FFI questionnaire associated with genetic variation of *5-HTTLPR*. This indicates that the genetic association effects with the ERP data oberved in adults are unlikely to be related to general differences in personality.

### Infants

**Figure 2:**
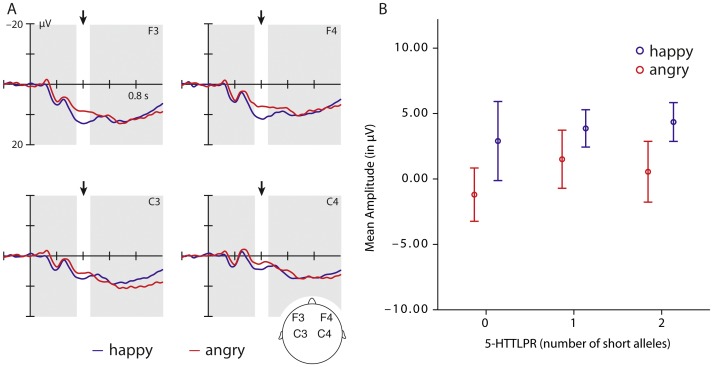
This figure shows the grand-average of the ERP responses to happy (blue) and angry (red) tone of voice at frontal and central electrodes in infants. (A) The graph (B) on the right side shows the mean amplitude of the ERP response to happy (blue) and angry (red) voices between 350 and 450 ms according to the genotype groups.

As shown in Figure 2A, the ERP analysis performed on the grand average data for the infant group revealed that angry voices elicited a more negative-going waveform when compared to happy voices, already between 350 and 450 ms, (*F* [1], [47] = 4.52, *p = *0.039, partial *η^2^* = 0.088), that is, earlier than for adults. This difference had its maximum at anterior electrode sites. For this time window there was no interaction between the factors emotion and *5-HTTLPR* genotype (see Figure 1B). This time window reflects perceptual effects in infants for whom even early obligatory ERP components are delayed compared to adults [28], [33]. There were no ERP differences between emotions during later time windows. It is important to note that it is unlikely that the absence of a genetic association effect in infants is due to the relatively small sample size for a genetic association study. This is because in prior work with the same infant sample [24], we found systematic differences in the ERP responses to facial expressions of emotion associated with the *5-HTTLPR* genotype.

There were no effects of gender, neither for the adults nor for the infants.

## Discussion

In the current study, we examined the effects of serotonin transporter genetic variation *(5-HTTLPR)* on the processing of emotional voice processing in adults and infants. The results revealed that in adults *5-HTTLPR* variation was associated with differences in the brain responses to emotional tone of voice. This is the first study that goes beyond facial expressions and visually presented emotionally valenced material [14], [15], [17], by showing that genetic variation at this locus affects vocal emotion processing. This suggests that genetic variation impacts emotion processing across face and voice (across audition and vision), contributing to individual differences in emotion perception in the adult in a general multisensory fashion. This notion is in correspondence with accounts that emphasize the role of shared multisensory processes in emotion recognition as evident in a considerable overlap in the brain systems involved in emotion recognition from face and voice [for review, see 38].

Our adult findings show that in adults *5-HTTLPR* variation was associated with differences in the ERP responses to emotional tone of voice over anterior brain regions (frontal and central electrodes) starting around 400 ms after voice onset (N400). This late ERP component is thought to reflect evaluative (cognitive) processes related to the recognition of emotion conveyed through the voice [11]. More specifically, it is thought that a more negative-going deflection (amplitude) of this ERP component indexes the effect of a more effortful and cognitively demanding processing of a stimulus, while a more positive-going deflection (amplitude) indexes the effect of facilitated processing of a stimulus [29]. The grand average analysis of the ERP data revealed that adults in general showed a more negative-going response to angry voices when compared to happy voices, suggesting that, in line with prior work on processing facial expressions of anger [39], processing angry voices demands more cognitive effort or control than processing happy voices. This provides support for the notion of a negativity bias, according to which negative stimuli are hypothesized to carry greater informational value than positive stimuli and thus require greater attention and cognitive processing [40], [41].

While angry voices when compared to happy voices had this general effect, there were critical differences in the ERP responses associated with variation in *5-HTTLPR* in adults. Our results revealed that carriers of the short allele of *5-HTTLPR* showed a decreased differentiation in the ERP responses elicited by happy and angry tone of voice, indicating that genetic variation impacts the way in which positive and negative emotional information in the voice is differentiated and evaluated. Specifically, the number of short alleles was associated with a linear decrease (more negative-going) in the amplitude of this ERP component in response to a happy tone of voice, suggesting that the facilitatory effects of processing positive (happy) voices were reduced for individuals carrying the short allele and facilitation was strongest for individuals homozygous for the long allele. Furthermore, there was a non-linear association (inverted u-shape) between the number of short alleles and the amplitude of this ERP component in response to angry voices, with individuals homozygous for either the short or the long allele showing the most negative-going amplitudes, suggesting greatest cognitive effort in the homozygous groups when processing angry voices. Taken together, this pattern of findings is in line with recent behavioral work showing that while affectively positive visual stimuli had the most facilitating effects on selective visual attention, affectively negative stimuli had the most slowing effects on selective attention in individuals homozygous for the long allele [35]. This suggests that genetic variation of *5-HTTLPR* is critically associated with the way in which positive and negative emotions differentially bias information processing. Such differential sensitivity to emotional information in individuals is thought to have far-reaching effects on daily emotional experiences and general well-being [42].

With regard to the association of genetic variation in *5-HTTLPR* with emotional voice processing across development, we had tested between three hypotheses: genetic variation is associated with differences (*i*) in early perceptual processing in infants, (*ii*) in later cognitive processing in adults, and (*iii*) in both early perceptual processing in infants and later cognitive processing in adults. Our findings showed that 7-month-old infants’ brain responses indicated that they can discriminate between emotions carried by the human voice as reflected in the early perceptual ERP effect. However, no late cognitive N400-like effect as a function of emotional valence could be observed for the infants. This was expected on the basis of prior language work which revealed no semantic N400 effect before the second year of life [43]. Thus, supporting the second hypothesis, the present data show that genetic variation is associated with cognitive processes of emotional evaluation which only emerge over development. In this context it is also important to note that these findings imply that only through experience with language the genetic association effect emerges, suggesting that adults would not have shown a similar genetic association effect when presented with foreign language stimuli. However, this remains to be studied in future work.

For our interpretion of the differences between infants and adults in the genetic association oberved in emotional voice processing, it is important to note that prior work with the same group of infants at the same age showed that genetic variation in *5-HTTLPR* was associated with differences in infants’ brain responses to facial expressions [24]. This indicates that infants’ differential sensitivity to facial expressions is impacted by genetic variation in *5-HTTLPR* while this is not the case for emotional voice processing. This might have to do with the fact that emotional facial expressions and other nonverbal means of emotional communication provide more powerful and effective means of communicating emotions from an evolutionary perspective than emotional tone of voice that needs to be extracted from speech [4], [44], [45]. Another contributing factor might be that while infants are able to produce the relevant facial expressions, they are not yet able to produce speech as used here [46], nor do they show semantic N400 effects and that this may hence preclude any differential effects in the current study. There is substantial evidence pointing towards a relation between perception and production in early social-cognitive development [47]. For example, there is some evidence from prior work in 7-month-old infants suggesting that variation in *5-HTTLPR* is associated with differences in the frequency of producing emotional expressions in daily life, in particular positive expressions such as smiling [24]. Specifically, 7-month-old infants that were homozygous for the short allele smiled significantly less and also responded less sensitively as indexed by their ERP responses to happy (smiling) facial expressions, suggesting a link between the production and perception of positive facial expressions. It might therefore be useful to extend the current approach in future work by examining infants’ brain responses to vocal expressions that are in their expressive repertoire such as laughing and crying [48], as this would provide a clearer comparison between vocal and facial emotional expressions. Notwithstanding these critical considerations regarding the exact nature of the genetic association effect across the visual and auditory domain, the current results shed important light on the developmental changes in the auditory domain that occur in how genetic variation in *5-HTTLPR* contributes to differences in emotional voice processing.

In line with our predictions based on prior work [32], [49], we found that 7-month-old infants discriminated between angry and happy tone of voice. Similar to adults angry voices elicited a more negative-going ERP response than happy voices. However, different from adults, infants showed earlier ERP differences distinguishing between the two emotions, while the adult ERP data (N400) reflect an evaluative (cognitive) process, the infant data appear to reflect differences in familiarity-based processes with more positive-going deflections indexing increased familiarity with an auditory stimulus [33], [50]. The result that happy voices elicited more positive-going ERP responses than angry voices might thus indicate that infants experience positive tone of voice as more familiar. Indeed, there is an extensive body of work showing that parents and adults across cultures use a specific form of speech called infant-directed speech or motherese when talking to infants that is chararterized by a happy tone of voice [51]–[54], making it the most familiar tone of voice for infants. Recently, it has been shown that infant-directed speech when compared to adult-directed speech results in similar ERP modulations (infant P350) as observed in the current study [55]. Taken together, the infant and adult ERP data suggest that adults differentiate between emotions during later processing stages (N400), reflecting semantic-level processes associated with the cognitive evaluation of the meaning of emotions [11], whereas infants distinguish between emotions during earlier processing stages (infant P350/adult P200), reflecting familiarity-based preceptual level processes engaged when making this distinction [33]. This finding supports the notion that the brain processes involved in distinuguishing between emotions communicated through speech prosody undergo development between infancy and adulthood.

In conclusion, to our knowledge this is the first study that systematically compared how genetic variation is associated with infants’ and adults’ brain responses to emotional information carried in the voice. Taking such a genetic imaging approach has been shown to be of great value for the understanding of individual differences in adults, and studying the association of genetic variation with brain responses as intermediate phenotypes, or so-called endophenotypes, has been argued to be a more powerful approach than studying gene effects on behavior (or personality traits) (Goldberg & Weinberger, 2004). Using this approach to compare adults and infants in the current study has revealed novel insights into our understanding of emotional voice processing by adding a developmental dimension to the complex picture of how genetic variation may affect human emotional sensitivity. The finding that the association between *5-HTTLPR* variation and differences in emotional voice processing emerges during development and is not present in infancy raises interesting hypotheses about how genetic variation may bias specific brain processes during development and thereby give rise to individual differences that ultimately contribute to complex phenotypes such as temperament and personality.
